# How Beneficial Is Pretraining on a Narrow Domain-Specific Corpus for Information Extraction about Photocatalytic Water Splitting?

**DOI:** 10.1021/acs.jcim.4c00063

**Published:** 2024-03-28

**Authors:** Taketomo Isazawa, Jacqueline M. Cole

**Affiliations:** † Cavendish Laboratory, Department of Physics, University of Cambridge, J. J. Thomson Avenue, Cambridge CB3 0HE, U.K.; ‡ ISIS Neutron and Muon Source, STFC Rutherford Appleton Laboratory, Harwell Science and Innovation Campus, Didcot, Oxfordshire OX11 0QX, U.K.

## Abstract

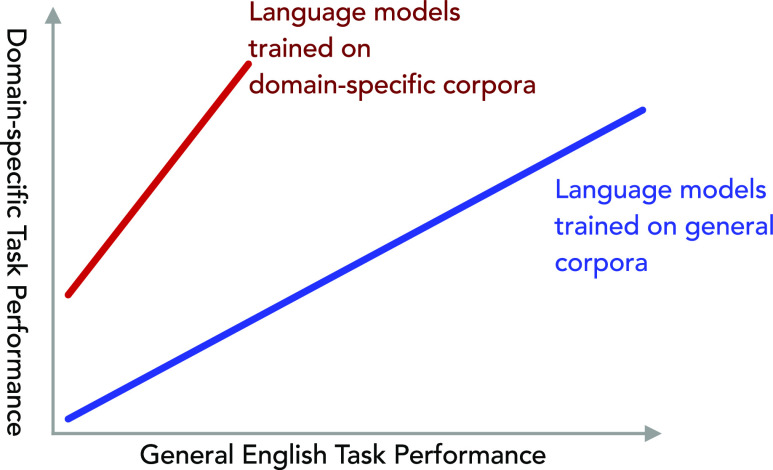

Language models trained on domain-specific corpora have been employed to increase the performance in specialized tasks. However, little previous work has been reported on how specific a “domain-specific” corpus should be. Here, we test a number of language models trained on varyingly specific corpora by employing them in the task of extracting information from photocatalytic water splitting. We find that more specific corpora can benefit performance on downstream tasks. Furthermore, PhotocatalysisBERT, a pretrained model from scratch on scientific papers on photocatalytic water splitting, demonstrates improved performance over previous work in associating the correct photocatalyst with the correct photocatalytic activity during information extraction, achieving a precision of 60.8(+11.5)% and a recall of 37.2(+4.5)%.

## Introduction

There has been a recent surge in domain-specific language models^1−4^ being trained with the aim of increasing the performance of language models on downstream tasks in that domain. In particular, there have been several reports of language models that have been pretrained on narrow domains within the field of materials science, such as battery materials^2^ or optical materials,^4^ that demonstrate superior performance over language models that have been trained on the materials science as a whole^5^ or on the broader scope of science.^1^ While research has shown that language models pretrained on smaller in-domain corpora can have improved performance on such tasks over a language model pretrained on a larger general corpus,^6^ this does not answer the question of what level of specificity is necessary for an “in-domain corpus”. For example, should there be pretrained language models for each downstream domain, or would a language model trained only on the physical sciences suffice for materials-science applications?

We will answer this question by comparing language models that have been pretrained on corpora of varying specificity in terms of their performance levels on information extraction from a specific domain, photocatalytic water splitting. Their ability to extract information is determined by using multiturn question answering^7^ as the downstream task since this approach has previously been used successfully to extract chemical data.^8^ The specific domain was chosen according to its topical promise of environmentally friendly energy from photocatalytic water splitting, the relative paucity of structured data for this domain, and the particularly high complexity of its knowledge representation due to the number of factors involved in determining photocatalytic activity. This high complexity of the knowledge representation results in information on multiple photocatalysts being represented in a single sentence or the information on a single photocatalyst being scattered across sentences. Consequently, this topic poses a stringent test for information extraction. Moreover, we have recently automated the construction of a database for this specific materials domain,^9^ creating rules that exploit inter- and intrasentence relations to associate photocatalytic activities and reaction conditions with their corresponding photocatalysts. Thus, we can use this database as a baseline for this work.

We will show that in-domain corpora can improve the performance of language models on downstream tasks, with the improvements being greatest when performing information extraction for very domain-specific chemical attributes, such as the cocatalysts associated with photocatalytic water splitting. When using the best available language model, the multiturn question-answering approach improves on previous work for photocatalytic data extraction, especially in terms of extracting the correct photocatalyst, where a precision of 60.8(+11.5)% and a recall of 37.2(+4.5)% is achieved. We focus our work on language models from the bidirectional encoder representations from transformers (BERT) family since these are open source and thus have been already adopted by the field of materials science for performing domain-specific tasks (e.g., MatSciBERT,^5^ BatteryBERT,^2^ and OpticalBERT^4^). Open-source implementations of all algorithms described in this paper have been made available on GitHub.

## Methods

### Data Sources for Our Language Models

To test the benefits of creating more domain-specific BERT-based models, we created two new language models by pretraining BERT-base^10^ using the same number of parameters in the same configuration as originally described, from scratch using two different textual corpora.

One corpus consists exclusively of academic papers pertaining to photocatalysis. These papers were scraped from two scientific academic publishers [Elsevier and the Royal Society of Chemistry (RSC)] using our “chemistry-aware” text-mining tool, ChemDataExtractor.^9,11,12^ The papers were selected by searching for the phrases “photocatalytic water splitting activity” and “photocatalyst hydrogen water activity”. This resulted in a corpus with 251,205 papers from Elsevier and 102,797 papers from RSC. Further details of this data selection process are given in the Supporting Information. This process generated a corpus of size 11 GB, which prior work suggests is enough to reap the benefits of in-domain corpora.^6^

The other corpus comprises a subset of the S2ORC corpus that represents the physical sciences as a whole.^13^ This corpus comprising the physical-sciences subset of S2ORC was created by selecting papers from the S2ORC corpus that were tagged as pertaining to the fields of physics, chemistry, and materials science. This generated a corpus of 57 GB, a much larger corpus than the aforementioned photocatalysis-specific corpus. As a point of wider comparison, the original BERT models were trained on a corpus of size 13GB^10^ while subsequently developed variants such as RoBERTa have used corpora as large as 160 GB.^14^

### Tokenizing the Corpora

Following the approach described in the original BERT paper,^10^ we trained WordPiece^15^ tokenizers with a 30,000 token vocabulary on each corpus. We used the HuggingFace tokenizers library^16^ to do this in practice and opted for the uncased variant in all cases.

By analyzing the tokenizers generated through this process, we gained insights into the differences between the corpora by comparing the overlap between vocabularies in the tokenizers for our collected corpora and that of BERT-base, as well as the tokenizers from other domain-specific BERT models, such as BatteryOnlyBERT^2^ and OpticalPureBERT.^4^ The BatteryOnlyBERT tokenizer was trained on a corpus consisting of papers scraped from the academic literature using ChemDataExtractor that contained the keyword “battery” from the RSC, Elsevier, and Springer publishers,^17^ while OpticalPureBERT had been trained on a corpus that had similarly been produced by scraping papers from the RSC and Elsevier using the keyword “optical material”.

**Figure 1:**
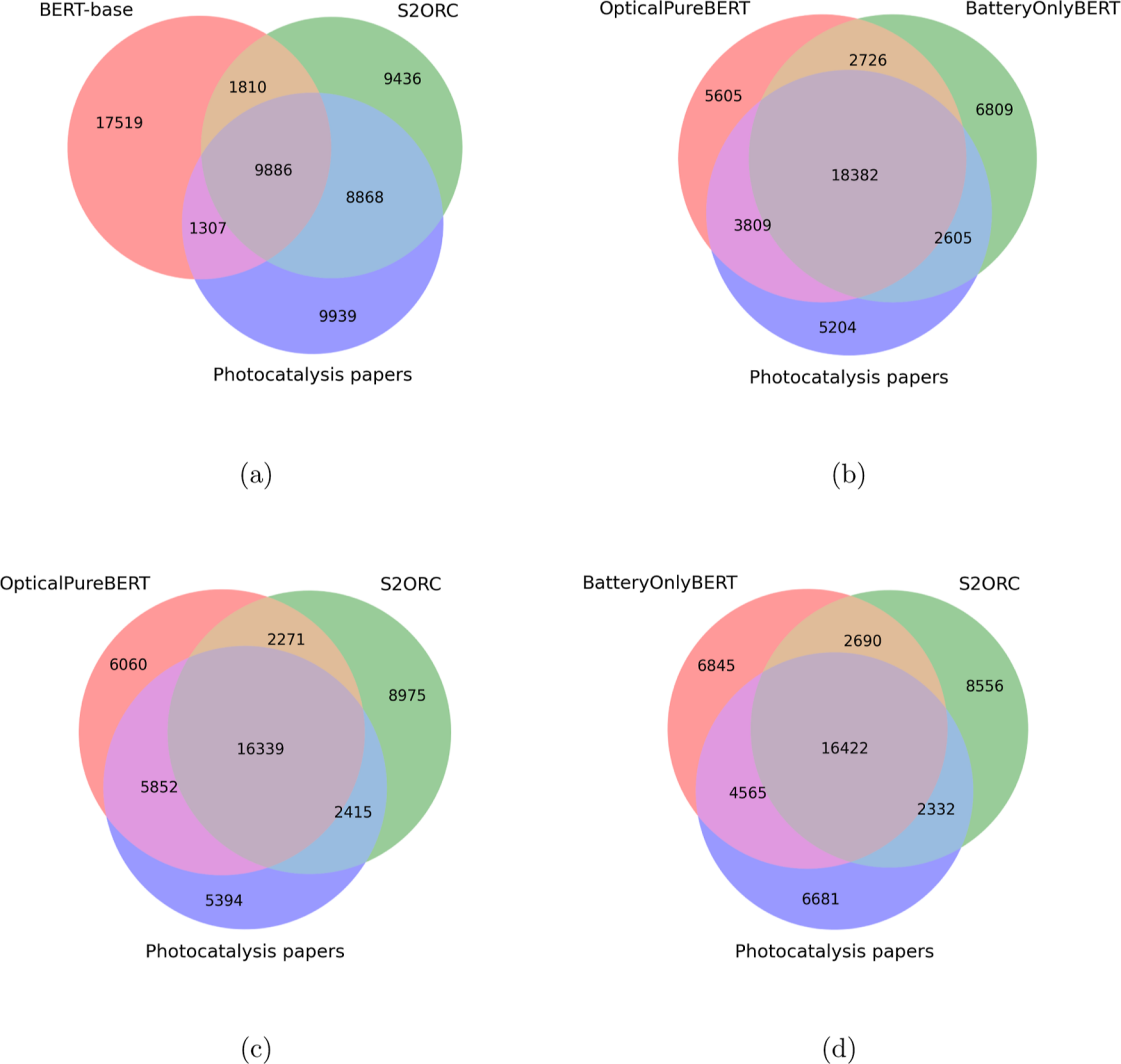
Venn diagrams comparing vocabularies of WordPiece tokenizers trained on different corpora. Each diagram shows comparisons between the vocabularies of (a) BERT-base, a tokenizer trained on photocatalysis papers, and a tokenizer trained on the S2ORC corpus filtered for physical sciences, (b) tokenizers trained on photocatalysis papers, papers mentioning optical properties (OpticalPureBERT), and papers mentioning batteries (BatteryOnlyBERT), (c) OpticalPureBERT, a tokenizer trained on photocatalysis papers, and a tokenizer trained on the aforementioned filtered S2ORC corpus, (d) BatteryOnlyBERT, a tokenizer trained on photocatalysis papers, and a tokenizer trained on the aforementioned filtered S2ORC corpus. Note the large areas of overlap between the tokenizer trained on photocatalysis papers and the tokenizer trained on text from other domains within materials science and the comparatively small overlap with the BERT-base tokenizer.

As can be seen in Figure 1, the vocabulary for photocatalysis papers is substantially different from that of the BERT base, but it significantly overlaps with the S2ORC data set, as would be expected given that photocatalysis is a subject of the physical sciences. Interestingly, even though the OpticalPureBERT and BatteryBERT models had been trained on corpora that were assembled from papers that did not pertain to photocatalysis, the vocabularies of the tokenizers overlap considerably not only with the S2ORC data set but also with the tokenizer trained purely on photocatalysis papers. This demonstrates a strong similarity in the vocabulary used in these different fields of materials science.

### Pretraining the Language Models

Two models that we obtain by pretraining the BERT-base architecture on the above corpora are referred to as PhotocatalysisBERT and PhysicalSciencesBERT, respectively, from here on. They represent two contrasting options: a very task-specific language model trained on a specialized but small corpus and a slightly less task-specific model trained on a less specialized but larger corpus.

Any systematic biases that may arise from the effects of training on each corpus were isolated by pretraining using the same number of parameters in the same configuration as the original BERT-base model.^10^ Furthermore, we ensured that papers that were used later for assessing data-extraction performance for either model were not included in the pretraining corpora, as that could also skew the results.

The HuggingFace transformers^18^ implementation of the BERT-base model was pretrained on the masked language modeling task using the Polaris supercomputing cluster at the Argonne Leadership Computing Facility (ALCF), Illinois, USA. Polaris contains NVIDIA A100 GPUs, and training was distributed across multiple nodes using DeepSpeed.^19^ A batch size of 2048 was used for effective training. More detail on the pretraining parameters can be found in the Supporting Information.

### Fine-Tuning the Language Models

The models were then fine-tuned so that they could be used for the downstream task of question answering to extract information about photocatalysis. The models were therefore fine-tuned for extractive question answering on the Stanford question-answering (SQuAD) v2.0 data set.^20^ The SQuAD v2.0 data set contains 150,000 crowd-sourced questions, the contexts in which the answer can be found, and the location of the answer within the context. Of these questions, 50,000 are “unanswerable”, i.e., the answers to these questions are not present in the contexts.

We followed the original BERT paper^10^ in approaching this task, where the context and question are concatenated, and the location of the answer is predicted by finding the token positions *i* and *j* (*i* ≤ *j*) that have the highest dot product with start and end vectors that are trained during fine-tuning.

In addition to fine-tuning the PhysicalSciencesBERT and PhotocatalysisBERT models on this task, we also used other readily available fine-tuned models and fine-tuned other language models to compare their performance. All results from fine-tuning these models on SQuAD v2.0 are presented in the Supporting Information.

These fine-tuned BERT models were then used to construct a photocatalysis knowledge base using a multiturn question-answering approach.^7,8^ This approach uses a series of SQuAD-style questions, each detecting spans within the text, to extract information from natural language.

For example, consider the following passage:

“Nanoferrite is emerging as a promising photocatalyst with a hydrogen evolution rate of 8.275 μmol h^–1^ and a hydrogen yield of 8275 μmol h^–1^ g^–1^ under visible light compared to 0.0046 μmol h^–1^ for commercial iron oxide (tested under similar experimental conditions).”^21^

It is relatively trivial to identify, with high precision, photocatalytic-activity-related values such as 8.275 μmol h^–1^ using simple rules based on the expected units used for this property. We can then find the corresponding material that relates to this property value by asking a conditional question given this prior knowledge about the property question “What photocatalytic material has a hydrogen evolution rate of 8.275 μmol h^–1^?” to find the corresponding material (in this case, nanoferrite). We can also ask follow-up questions such as “What co-catalytic material was used alongside nanoferrite when measuring the hydrogen evolution rate to be 8.275 μmol h^–1^?” to extract any associated cocatalysts; in this case, there is no information on this in the passage, which the model should be able to identify due to its fine-tuning on SQuAD v2.0.

This approach is similar to other methods that use generative LLMs to generate structured data from unstructured text.^22,23^ However, a key difference is that the extractions from a multiturn question-answering approach are always grounded in the text, as models trained on the SQuAD v2.0 task will output spans within the text where the model predicted the answer to the question to be. This is in contrast to the aforementioned methods that use generative LLMs, where the LLM generates text corresponding to the data it believes is in the text, and it is not straightforward to know if the values are hallucinated.

**Figure 2:**
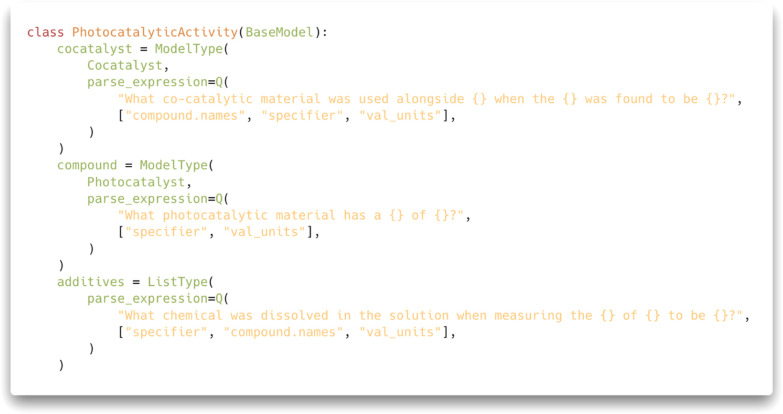
Simplified version of the knowledge representation used in ChemDataExtractor for this study, showing how multiturn question answering can be set up in practice to extract material properties. The specifier refers to the property name identified in the sentence via rule-based parsing (e.g., “photocatalytic activity”). For the sake of brevity, we have removed some fields and implementation details that were not relevant to multiturn question answering.

We implemented this algorithm using the chemistry-aware text-mining tool ChemDataExtractor, as shown in Figure 2. As shown in the example, the user can bind question templates with the necessary knowledge representation in ChemDataExtractor. In this way, multiturn question answering can be integrated into more classical extraction methods, such as rule-based extraction, which we can use in tandem to identify property attributes such as values and units with high precision and recall. This model can then be used to extract photocatalytic information.

### Evaluating the Quality of Information Extraction

We assessed the quality of information extraction via this multiturn question-answering method using 330 manually labeled open-access papers, of which 220 were published by Elsevier and 110 were published by the RSC from our previous work.^9^ We followed the methodology used in this previous work to calculate the precision and recall of this data set, adapting the data set slightly to account for a change in knowledge representation.

In addition to the two models we pretrained (PhotocatalysisBERT and PhysicalSciencesBERT), we also evaluated the performance levels of a number of off-the-shelf models including BERT-base,^10^ MiniLM,^24^ DeBERTaV3,^25^ BatteryBERT,^2^ and OpticalBERT^4^ on this multiturn question-answering task for information extraction.

## Results and Discussion

### Technical Validation

PhotocatalysisBERT and PhysicalSciencesBERT models were both found to perform at a similar level to the original BERT-base-uncased model on the SQuAD v2.0 question-answering data set,^10,26^ achieving a score of 70.3 and 69.7, respectively, on the SQuAD v2.0 development set, compared to 73.4 for BERT-base-uncased,^26^ on the exact match scoring metric. The exact match metric measures the percentage of predictions that exactly match one of the answers provided in the ground truth data set. The performance levels, as measured by the precision, recall, and F1 scores of both of these models on the photocatalytic data-extraction task, are shown in Table 1, alongside those from the baseline and our previous rule-based data-extraction approach (DepIE) within ChemDataExtractor.

**Table 1:** Precision, Recall, and F1-Score (All Percentages, Range 0–100) Calculated by Exact Matches on Our Corpus of 330 Hand-Labeled Open-Access Papers for PhotocatalysisBERT and PhysicalSciencesBERT, Compared against Our Baseline^a^

	PhotocatalysisBERT			PhysicalSciencesBERT			DepIE (baseline)		
	precision	recall	F1	precision	recall	F1	precision	recall	F1
**overall**	**68.2**	**38.5**	**49.2**	62.0	37.7	46.9	67.8	36.8	47.8
activity value/units	87.9	45.8	60.2	89.2	46.5	61.1	**91.8**	**47.2**	**62.3**
photocatalyst name	**60.8**	**37.2**	**50.7**	52.7	34.2	41.5	49.3	32.7	39.3
co-catalyst name	46.7	**47.7**	47.2	36.5	48.7	41.8	**48.8**	47.6	**48.2**
additive name	69.4	**27.9**	39.8	62.3	27.1	37.7	**77.5**	25.0	**37.8**

a The highlighted values represent the best score under each metric (Precision, Recall, and F1-score) for each extraction task (overall extraction, activity value/units, photocatalyst name, etc.). We have omitted irradiation time and light source from our assessment as we did not use multiturn question answering to extract these but rather used the same rule-based approach as with the baseline. As a result, the overall score for precision, recall, and F1 has been recalculated for the baseline from the original paper.^9^

The results in Table 1 show that the PhotocatalysisBERT model is significantly ahead of both the baseline (DepIE) and the PhysicalSciencesBERT model in its data-extraction performance, especially when it comes to extracting the photocatalyst name. This result shows the benefits of using question answering as well as the benefit of training with a domain-specific corpus, even if it is substantially smaller than a more general corpus that contains the domain knowledge. At the same time, some shortcomings can be seen from the multiturn question-answering approach given its performance on cocatalyst names and additive names according to the results in Table 1. A possible explanation for this shortfall, based on a closer look at the false positives identified by the system as cocatalyst or additive names, is the fact that these chemical names are often written in the text of the document far away from the sentence containing information on photocatalytic activity. This occurs often when the activity is stated in the results section and the cocatalyst or additive in the methods section. Given the models were fine-tuned on SQuAD v2.0, where the maximum context given to the language model for question answering is one paragraph, we match this in our multiturn question answering. As a result, the language models completely lack any contextual relationship between the photocatalyst name and that of a cocatalyst or additive and cannot possibly identify these relationships.

Another possible reason for the lower performance levels on identifying cocatalyst and additive names is the fact that we extract these fields as follow-up questions (e.g., “*What co-catalytic material was used alongside* TiO_2_*when the approximate quantum yield was found to be 3%?*”). The sequential nature of this multiturn question-answering approach means that its performance level necessarily depends upon the photocatalyst name being correct when the data are extracted via multiturn question answering. As a competing factor, performance levels are slightly boosted above those of a naïve approach where cocatalysts and additives are either ignored or merged in at random as the information can be merged in through ChemDataExtractor’s interdependency resolution which takes into account the proximity of the extractions and the model’s confidence.^9,11^

### Assessing the Benefit of a Domain-Specific Corpus

Based on the evaluation results for these two new BERT models, we can see the value of a language model trained on a more specific domain for information extraction, but we also wanted to see how this compared to a wider range of pretrained models. To test this, we looked at the performance levels of other BERT-based models, both those trained on materials science-specific corpora and general language corpora, on this photocatalytic information-extraction task and compared this with their performance levels on the SQuAD v2.0 data set.

The design logic behind this test comes from the following premise: as multiturn question answering converts a data-extraction task into a sequential series of question-answering tasks, under this setting, a general-question-answering task and the information-extraction task are technically equivalent. As we are doing question analysis either way, we would expect the performance level of a fine-tuned language model answering general English questions from the SQuAD v2.0 development data set to correlate well with the performance level on extracting photocatalytic data using the multiturn question-answering method. However, a deviation from this general trend correlation would indicate that there may be some extra benefit from the use of a domain-specific corpus in pretraining.

**Figure 3:**
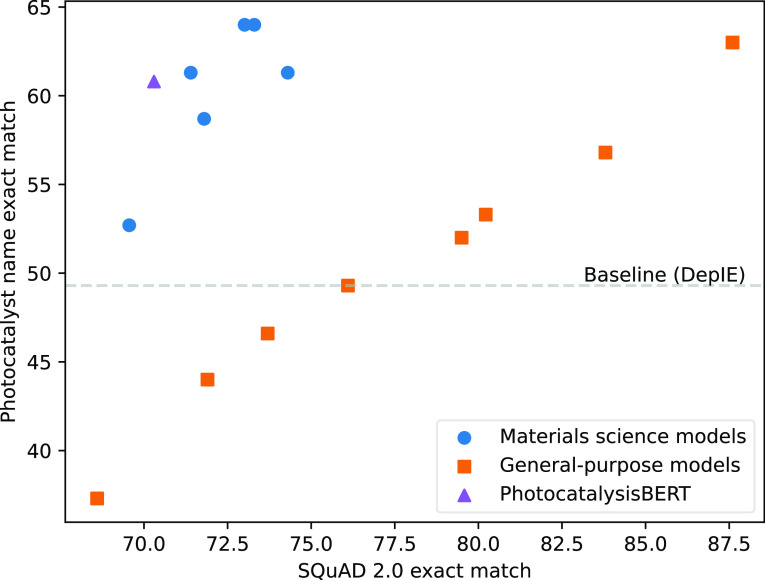
Performance levels of various BERT models on extracting photocatalyst names with our multiturn question-answering approach, measured using our photocatalysis data corpus,^9^ plotted against performance levels on answering general English questions measured using the SQuAD v2.0 evaluation data set.^20^ All models were fine-tuned on the SQuAD v2.0 data set. Material science-specific models include PhysicalSciencesBERT, BatteryBERT, BatteryOnlyBERT,^2^ OpticalPureBERT,^4^ and MatSciBERT.^5^ Note their relatively high performance in identifying photocatalyst names compared to general-purpose models. For a full list of the models evaluated and their performance, see Supporting Information.

The result in terms of the precision of such models in extracting the photocatalyst name using multiturn question answering is plotted against their performance level on the development set of the SQuAD v2.0 data set in Figure 3. Full results for all of the other evaluations for each of the models are provided in the Supporting Information.

In general, as expected, there is a strong correlation between the performance levels of language models on answering general English questions, as measured by their performance on the SQuAD v2.0 development set, and their performance levels on extracting the correct photocatalyst names, although we did not calculate any quantitative measures for this due to the relatively small sample size. Remarkably, we can also see that the models trained on domain-specific corpora from materials science seem to have a different gradient and offset, forming a group that, for any given exact match score on SQuAD v2.0 in the range explored, consistently outperforms those language models that have been trained on a general purpose corpus. In fact, the benefit of a language model being pretrained on materials-science papers seems to be so great that, when extracting photocatalyst names, these language models can outperform those that are much larger and perform much better on SQuAD v2.0, such as the large version of DeBERTa v3^25,27^—the top-performing language model that we tested on SQuAD v2.0. Within this group, while the PhotocatalysisBERT model performs well, it does not seem to particularly outperform those trained on other, less domain-specific corpora.

**Figure 4:**
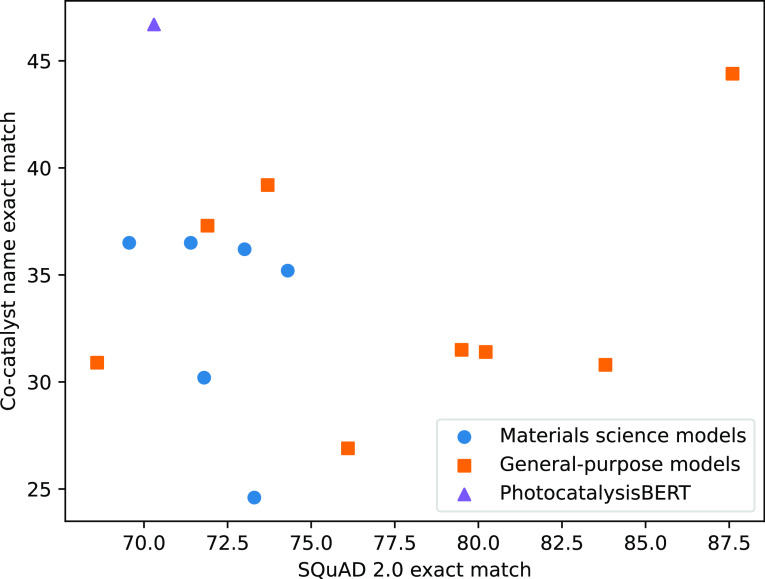
Performance levels of various BERT models on extracting cocatalyst names with our multiturn question-answering approach, measured using our photocatalysis data corpus,^9^ plotted against performance levels on answering general English questions measured using the SQuAD v2.0 evaluation data set.^20^ All models were fine-tuned on the SQuAD v2.0 data set. Material science-specific models include PhysicalSciencesBERT, BatteryBERT, BatteryOnlyBERT,^2^ OpticalPureBERT,^4^ and MatSciBERT.^5^ The highest performing general-purpose model was DeBERTa v3 large. For a full list of the models evaluated and their performance, see Supporting Information.

However, when considering the extraction of auxiliary properties, such as the cocatalyst name, the story is completely different, as shown in Figure 4. Despite other language models performing far better in answering general English questions from SQuAD v2.0, PhotocatalysisBERT outperforms every other model in extracting the correct cocatalyst names. While it is difficult to draw definitive conclusions, the ranking within language models that were pretrained on materials science models offers some insights. Their performance levels, in terms of precision on an exact match with the cocatalyst name in the human-annotated data, follow the order (highest first): PhotocatalysisBERT(46.7) > PhysicalSciencesBERT(36.5) > MatSciBERT(36.5) > OpticalPureBERT(36.2) > OpticalBERT(35.2) > BatteryOnlyBERT(30.2) > BatteryBERT(24.6). Based on these results, it is possible to see that pretraining language models on photocatalysis-specific corpora yields particularly significant benefits and that those language models that have been pretrained solely on corpora from the physical sciences perform better than equivalent models where continued pretraining was performed on top of a BERT-base model.

While this comparison is again more qualitative than quantitative due to the small number of language models that were compared, this indicates the benefit of pretraining on very specific corpora for certain tasks. The strong benefit of a domain-specific corpus in extracting cocatalyst names stands in contrast to the aforementioned trend seen in extracting photocatalyst names. In the latter case, there was a general correlation between the SQuAD v2.0 performance and the data-extraction performance, with a boost being observed if a materials-science-based corpus was used during pretraining but no clear benefit to a very specialized corpus.

A possible explanation for the difference between the trends in performance of the language models when extracting photocatalyst names and extracting cocatalyst names is the difference in the extent to which the two properties are niche. The concept of photocatalysts being associated with some activity is perhaps something straightforward that does not require much expert knowledge beyond the knowledge of the existence of catalysts, which is surely contained in corpora in materials science. In contrast, perhaps a more specific training corpus, or one that contains more information on the subject field of materials science, is required to understand the existence of cocatalysts and what types of chemicals are feasible. This explains both the high performance of the PhotocatalysisBERT model displayed in Figures 3 and 4 and the poor performance of models where pretraining was continued from the original BERT base model, as in that case, the sentences seen throughout the course of training were, on average, less specific to the physical sciences. Outside of models pretrained on materials science corpora, the large version of DeBERTa v3^25,27^ again performed best.

## Conclusions and Future Work

This work presents two key results. First, it presents the PhotocatalysisBERT and PhysicalSciencesBERT models, which are BERT-like models that have been pretrained on corpora focused on photocatalytic water splitting and the physical sciences, respectively. These were used to extract information on photocatalytic water splitting, and they demonstrate enhanced performance over previous work.^9^ In particular, their performance was superior in identifying the correct photocatalyst associated with a given photocatalytic activity, resulting in an 11.2 percentage-point increase in precision and a 4.5 percentage-point increase in recall for the PhotocatalysisBERT model. This realistic downstream use of language models for information extraction in materials science demonstrates the viability of multiturn question answering^7^ as a replacement for previous information-extraction methods and its potential in creating a database on photocatalytic water-splitting.

The second key result of this paper is the comparison of multiple language models that have been pretrained on corpora of varying levels of domain specificity in terms of their performance in information extraction for photocatalytic water splitting. We found that for “simpler” tasks, such as extracting the photocatalyst name that is associated with a certain photocatalytic activity, any language model that had been pretrained on a material-science corpus that we investigated seemed to offer just as much benefit as a photocatalysis-specific corpus. In contrast, for more specialized tasks such as extracting the cocatalyst that is associated with a photocatalyst, the closer the pretraining corpus of a language model was to the photocatalytic-water-splitting domain, the higher the performance seemed to be, with continued pretraining performing worse than pretraining a language model from scratch. This work may thus help with corpus and language model selection for researchers working with specialized domains looking to pretrain language models for downstream tasks and the expected performance gains of doing so.

An important corollary to these findings is that one should consider the balance between such performance gains and the environmental cost of pretraining new models. The pretraining of a single BERT-base model is estimated to emit 1438 lbs of CO_2_, which is the same order of magnitude as the amount of CO_2_ emitted per passenger in a flight from New York to San Francisco (1984 lbs).^28^ Nonetheless, considering the higher performance of domain-specific models shown in this paper despite their often smaller size, it may make sense to pretrain a new model from scratch if the model is to be used widely.

Owing to the small number of language models tested (15 models, of which 7 were pretrained on material-science data sets and the rest on general-purpose data sets), it is difficult to draw fully quantitative results on this front. However, this would be an interesting avenue for future work. This could be accomplished artificially by taking a specific corpus like that used to train the PhotocatalysisBERT model and progressively diluting it with documents from a general-purpose corpus such as the BookCorpus,^29^ English Wikipedia, or the Pile.^30^

Another potential piece of future work could involve feeding in the entire document as context to an extractive question-answering model for the multiturn question-answering task instead of a few sentences. This could increase performance as, at times, the questions were impossible to answer due to the information being outside the context window. While this is beyond the scope of the models tested in this paper due to the lengths of sequences seen in pretraining and fine-tuning, it may be feasible and yield better results for newer, larger models. However, such models would come with further challenges in training for specialized domains, as larger models require more training data to perform well.^31^ Of course, larger models also pose more general challenges in terms of hardware, with a much higher computational cost-limiting accessibility and a substantial environmental cost due to their power consumption.

## Data Availability

All of the scripts used to process the data that were employed to pretrain PhotocatalysisBERT and PhysicalSciencesBERT are available online,^32^ as are the scripts used to extract and evaluate photocatalysis records,^33^ the knowledge representation used,^34^ and the adapted annotations used as a gold standard.^35^ Multiturn question answering is also available in the newest version (v2.2) of ChemDataExtractor,^36^ and the pretrained and fine-tuned models are available on the Molecular Engineering Group HuggingFace.^37^

## Supporting Information Available

Details on pretraining corpus, pretraining hyperparameters, and photocatalysis extraction results for all models tested (PDF)
