# Supplementary material for: How Beneficial Is Pretraining on a Narrow Domain-Specific Corpus for Information Extraction about Photocatalytic Water Splitting?
Source: J Chem Inf Model. 2024 Mar 28;64(8):3205–12. doi: 10.1021/acs.jcim.4c00063 (PMC11040717; doi:10.1021/acs.jcim.4c00063)
Supplement: Supplementary file 1 — ci4c00063_si_001.pdf [file ci4c00063_si_001.pdf]

Supporting Information
How beneficial is pre-training on a narrow domain-specific corpus for information extraction about photocatalytic water splitting?

Taketomo Isazawa[†] and Jacqueline M. Cole[*,†,‡]

†*Cavendish Laboratory, Department of Physics, University of Cambridge, J. J. Thomson Avenue, Cambridge, CB3 0HE, UK*

‡*ISIS Neutron and Muon Source, STFC Rutherford Appleton Laboratory, Harwell Science and Innovation Campus, Didcot, Oxfordshire, OX11 0QX, UK*

E-mail: jmc61@cam.ac.uk

Details on Pre-training Corpus

The pre-training corpus was gathered using ChemDataExtractor's scraping tools from the Royal Society of Chemistry and Elsevier papers. In Elsevier's case, this interfaced with their official API for scraping. For both publishers, the set of results from the following set of queries were used:

- photocatalytic water splitting
- photocatalyst water hydrogen
- catalysis water splitting

- hydrogen production catalyst
- catalysis hydrogen production
- water splitting hydrogen
- photocatalysis
- photocatalyst

Results were gathered for the years 2000 to 2023 for Elsevier. This resulted in a photocatalytic water splitting-specific corpus of size 11 GB being generated.

Pre-training Hyperparameters

To maintain comparability with the original BERT base model, the PhotocatalysisBERT and PhysicalSciencesBERT models were trained to have the same number of parameters, so we used WordPiece embeddings[1] with a 30,000 token vocabulary, the hidden size was 768, there were 12 attention heads, and there were 12 hidden layers, resulting in 110 million total parameters.

Other hyperparameters during training, which were kept equal between PhotocatalysisBERT and PhysicalSciencesBERT, are as follows:

- Optimiser type: AdamW
- Adam beta 1: 0.9
- Adam beta 2: 0.999
- Adam epsilon: 1e-8
- Attention dropout probability: 0.1
- FP16 training: true

- Hidden activation type: GELU
- Hidden dropout probability: 0.1
- Initialiser range: 0.02
- Intermediate size: 3072
- Layer norm epsilon: 1e-12
- Learning rate: 8e-5
- Learning rate scheduler type: linear
- Max position embeddings: 512
- Position embedding type: absolute
- Max grad norm: 1
- Max steps: 187500
- Transformers version: 4.25.1
- Number of warmup steps: 10000

The BERT models were trained on the Masked Language Modelling (MLM) task with the ALCF Polaris cluster containing NVIDIA A100 GPUs, with training distributed across multiple nodes using DeepSpeed[2]. A batch size of 2,048 was used, and they were pre-trained for 187,500 steps. The maximum sequence length was also kept at 512 tokens.

Photocatalysis Extraction Results for All Models

All language models listed in this work were assessed in the same way as the PhotocatalysisBERT and PhysicalSciencesBERT models were in the main paper, looking at their exact

match for our photocatalysis dataset[3]. SQuAD v2.0 performance levels quoted are for the development set.

Table S1: Precision and Recall on our photocatalysis dataset[3] for BatteryOnlyBERT and BatteryBERT[4], models pre-trained on battery corpora from scratch and with continued training from BERT-base weights. BatteryOnlyBERT achieved a SQuAD v2.0 exact match of 71.8 and F1 score of 75.7 on the development set. BatteryBERT achieved a SQuAD v2.0 exact match of 73.3 and F1 score of 77.0 on the development set. As discussed in the main text, BatteryOnlyBERT performs better when extracting co-catalysts, but for all other properties BatteryBERT performs better.

	BatteryOnlyBERT			BatteryBERT		
	Precision	Recall	F1	Precision	Recall	F1
**Overall**	62.3	37.6	46.9	63.7	38.8	48.2
Activity value/units	86.7	45.8	59.9	88.0	46.5	60.8
Photocatalyst name	58.7	36.7	45.1	64.0	39.0	48.5
Co-catalyst name	30.2	44.4	36.0	24.6	40.0	30.4
Additive name	65.3	26.7	37.9	71.4	29.2	41.4

Table S2: Precision and Recall on our photocatalysis dataset[3] for OpticalPureBERT and OpticalBERT[5], models pre-trained on optical material corpora from scratch and with continued training from BERT-base weights. OpticalPureBERT achieved a SQuAD v2.0 exact match of 73.0 and F1 score of 77.0 on the development set. OpticalBERT achieved a SQuAD v2.0 exact match of 74.3 and F1 score of 78.0 on the development set.

	OpticalPureBERT			OpticalBERT		
	Precision	Recall	F1	Precision	Recall	F1
**Overall**	67.9	39.2	49.7	65.6	38.7	48.7
Activity value/units	89.3	47.2	61.8	88.0	46.5	60.8
Photocatalyst name	64.0	39.3	48.7	61.3	38.0	46.9
Co-catalyst name	36.2	42.5	39.1	35.2	46.3	40.0
Additive name	71.4	28.7	40.9	71.7	27.5	39.7

Table S3: Precision and Recall on our photocatalysis dataset[3] for DeBERTa v3 base and DeBERTa v3 large[6–8]. DeBERTa v3 large achieved a SQuAD v2.0 exact match of 88.1 and F1 score of 91.2 on the development set. DeBERTa v3 base achieved a SQuAD v2.0 exact match of 83.8 and F1 score of 87.4 on the development set.

	DeBERTa v3 large			DeBERTa v3 base		
	Precision	Recall	F1	Precision	Recall	F1
**Overall**	66.8	37.5	48.1	63.2	37.1	46.8
Activity value/units	87.7	45.1	59.5	87.8	45.8	60.2
Photocatalyst name	63.0	37.4	46.9	56.8	35.6	43.8
Co-catalyst name	44.4	47.6	46.0	30.8	42.1	35.6
Additive name	62.0	25.4	36.1	70.2	27.1	39.1

Table S4: Precision and Recall on our photocatalysis dataset[3] for MatSciBERT[9]. The model achieved a SQuAD v2.0 exact match of 71.4 and F1 score of 75.2 on the development set.

	Precision	Recall	F1
**Overall**	66.7	39.1	49.2
Activity value/units	89.3	47.2	61.8
Photocatalyst name	61.3	38.3	47.1
Co-catalyst name	36.5	46.3	40.8
Additive name	72.3	27.9	40.2

Table S5: Precision and Recall on our photocatalysis dataset[3] for SciDeBERTa[10]. While trained on the same scientific corpus as PhysicalSciencesBERT (S2ORC[11]), this model was trained on trained on general scientific data instead of being restricted to the physical sciences, so was plotted on the graphs in the main paper as a general-purpose model, instead of a material science model. The model achieved a SQuAD v2.0 exact match of 80.2 and F1 score of 83.5 on the development set.

	Precision	Recall	F1
**Overall**	63.3	38.3	47.8
Activity value/units	89.3	47.2	61.8
Photocatalyst name	53.3	35.1	42.3
Co-catalyst name	31.4	43.2	36.4
Additive name	72.0	29.5	41.9

Table S6: Precision and Recall on our photocatalysis dataset[3] for BERT base uncased[12,13]. Being trained with similar hyperparameters, this model is the closest analogue to PhotocatalysisBERT and PhysicalSciencesBERT trained on a general purpose corpus. The model achieved a SQuAD v2.0 exact match of 73.7 and F1 score of 77.9 on the development set.

	Precision	Recall	F1
**Overall**	62.8	38.0	47.4
Activity value/units	88.0	46.5	60.8
Photocatalyst name	46.7	31.8	37.8
Co-catalyst name	39.2	48.8	43.5
Additive name	73.5	30.0	43.0

Table S7: Precision and Recall on our photocatalysis dataset[3] for XLM RoBERTa large[14,15], a cross-language model. The model achieved a SQuAD v2.0 exact match of 81.8 and F1 score of 84.9 on the development set.

	Precision	Recall	F1
**Overall**	61.8	37.4	46.6
Activity value/units	88.0	46.5	60.8
Photocatalyst name	52.0	34.2	41.3
Co-catalyst name	31.5	45.6	37.4
Additive name	70.2	27.1	39.1

Table S8: Precision and Recall on our photocatalysis dataset[3] for MiniLM[16,17], a model distilled from a BERT base-sized UniLM v2[18] model. The model achieved a SQuAD v2.0 exact match of 76.1 and F1 score of 79.5 on the development set.

	Precision	Recall	F1
**Overall**	59.4	36.2	45.0
Activity value/units	88.0	46.5	60.8
Photocatalyst name	49.3	33.0	39.6
Co-catalyst name	26.9	38.9	31.8
Additive name	65.3	26.2	37.4

Table S9: Precision and Recall on our photocatalysis dataset[3] for tinyBERT[19,20], another distilled model. The model achieved a SQuAD v2.0 exact match of 71.9 and F1 score of 76.4 on the development set.

	Precision	Recall	F1
**Overall**	61.6	37.5	46.6
Activity value/units	89.3	47.2	61.8
Photocatalyst name	44.0	30.8	36.3
Co-catalyst name	37.3	47.5	41.8
Additive name	71.4	28.7	41.0

Table S10: Precision and Recall on our photocatalysis dataset[3] for distilled BERT medium[21], where a BERT large model was used as the teacher model. The model achieved a SQuAD v2.0 exact match of 68.6 and F1 score of 72.8 on the development set.

	Precision	Recall	F1
**Overall**	57.5	36.1	44.4
Activity value/units	89.3	47.2	61.8
Photocatalyst name	37.3	27.5	31.6
Co-catalyst name	30.9	44.7	36.6
Additive name	69.4	27.9	39.8

References

(1) Wu, Y.; Schuster, M.; Chen, Z.; Le, Q. V.; Norouzi, M.; Macherey, W.; Krikun, M.; Cao, Y.; Gao, Q.; Macherey, K.; Klingner, J.; Shah, A.; Johnson, M.; Liu, X.; Kaiser, L.; Gouws, S.; Kato, Y.; Kudo, T.; Kazawa, H.; Stevens, K.; Kurian, G.; Patil, N.; Wang, W.; Young, C.; Smith, J.; Riesa, J.; Rudnick, A.; Vinyals, O.; Corrado, G.; Hughes, M.; Dean, J. Google's Neural Machine Translation System: Bridging the Gap between Human and Machine Translation. *CoRR* **2016**, *abs/1609.08144*.

(2) Rajbhandari, S.; Rasley, J.; Ruwase, O.; He, Y. ZeRO: Memory Optimizations Toward Training Trillion Parameter Models. SC20: International Conference for High Performance Computing, Networking, Storage and Analysis. 2020; pp 1–16.

(3) Isazawa, T.; Cole, J. M. Automated Construction of a Photocatalysis Database for Water-Splitting Applications by exploiting Inter- and Intra-Sentence relations. *Sci. Data* **2023**, *10*, 651.

(4) Huang, S.; Cole, J. M. BatteryBERT: A Pretrained Language Model for Battery Database Enhancement. *J. Chem. Inf. Model.* **2022**, *62*, 6365–6377.

(5) Zhao, J.; Huang, S.; Cole, J. M. OpticalBERT and OpticalTable-SQA: Text- and Table-Based Language Models for the Optical-Materials Domain. *J. Chem. Inf. Model.* **2023**, *63*, 1961–1981.

(6) He, P.; Gao, J.; Chen, W. DeBERTaV3: Improving DeBERTa using ELECTRA-Style Pre-Training with Gradient-Disentangled Embedding Sharing. *CoRR* **2021**, *abs/2111.09543*.

(7) deepset/deberta-v3-large-squad2 · Hugging Face — huggingface.co. `https://huggingface.co/deepset/deberta-v3-large-squad2`, [Accessed 24-Apr-2023].

(8) deepset/deberta-v3-base-squad2 · Hugging Face — huggingface.co. `https://huggingface.co/deepset/deberta-v3-base-squad2`, [Accessed 24-Apr-2023].

(9) Gupta, T.; Zaki, M.; Krishnan, N. A. MatSciBERT: A materials domain language model for text mining and information extraction. *npj Comput. Mater.* **2022**, *8*, 102.

(10) Jeong, Y.; Kim, E. SciDeBERTa: Learning DeBERTa for Science Technology Documents and Fine-Tuning Information Extraction Tasks. *IEEE Access* **2022**, *10*, 60805–60813.

(11) Lo, K.; Wang, L. L.; Neumann, M.; Kinney, R.; Weld, D. S2ORC: The Semantic Scholar Open Research Corpus. Proceedings of the 58th Annual Meeting of the Association for Computational Linguistics. Online, 2020; pp 4969–4983.

(12) Devlin, J.; Chang, M.; Lee, K.; Toutanova, K. BERT: Pre-training of Deep Bidirectional Transformers for Language Understanding. *CoRR* **2018**, *abs/1810.04805*.

(13) deepset/bert-base-uncased-squad2 · Hugging Face — huggingface.co. `https://huggingface.co/deepset/bert-base-uncased-squad2`, [Accessed 21-Apr-2023].

(14) Conneau, A.; Khandelwal, K.; Goyal, N.; Chaudhary, V.; Wenzek, G.; Guzmán, F.; Grave, E.; Ott, M.; Zettlemoyer, L.; Stoyanov, V. Unsupervised Cross-lingual Representation Learning at Scale. *CoRR* **2019**, *abs/1911.02116*.

(15) deepset/xlm-roberta-large-squad2 · Hugging Face — huggingface.co. `https://huggingface.co/deepset/xlm-roberta-large-squad2`, [Accessed 24-Apr-2023].

(16) Wang, W.; Wei, F.; Dong, L.; Bao, H.; Yang, N.; Zhou, M. MiniLM: Deep Self-Attention Distillation for Task-Agnostic Compression of Pre-Trained Transformers. *Advances in Neural Information Processing Systems* **2020**, *33*, 5776–5788.

(17) deepset/minilm-uncased-squad2 · Hugging Face — huggingface.co. `https://huggingface.co/deepset/minilm-uncased-squad2`, [Accessed 24-Apr-2023].

(18) Bao, H.; Dong, L.; Wei, F.; Wang, W.; Yang, N.; Liu, X.; Wang, Y.; Gao, J.; Piao, S.; Zhou, M.; others Unilmv2: Pseudo-masked language models for unified language model pre-training. International conference on machine learning. 2020; pp 642–652.

(19) Jiao, X.; Yin, Y.; Shang, L.; Jiang, X.; Chen, X.; Li, L.; Wang, F.; Liu, Q. TinyBERT: Distilling BERT for Natural Language Understanding. *CoRR* **2019**, *abs/1909.10351*.

(20) deepset/tinybert-6l-768d-squad2 · Hugging Face — huggingface.co. `https://huggingface.co/deepset/tinybert-6l-768d-squad2`, [Accessed 24-Apr-2023].

(21) deepset/bert-medium-squad2-distilled · Hugging Face — huggingface.co. `https://huggingface.co/deepset/bert-medium-squad2-distilled`, [Accessed 24-Apr-2023].
